# Influence of Chemical Kinetics on Predictions of Performance of Syngas Production From Fuel-Rich Combustion of CO_2_/CH_4_ Mixture in a Two-Layer Burner

**DOI:** 10.3389/fchem.2019.00902

**Published:** 2020-01-21

**Authors:** Junrui Shi, Mingming Mao, Houping Li, Yongqi Liu, Yang Liu, Yangbo Deng

**Affiliations:** ^1^School of Transportation and Vehicle Engineering, Shandong University of Technology, Zibo, China; ^2^Marine Engineering College, Dalian Maritime University, Dalian, China

**Keywords:** fuel-rich combustion, porous media, syngas production, numerical study, chemical kinetics

## Abstract

Numerical investigations on partial oxidation combustion of CO_2_/CH_4_ mixture were executed for a two-layer burner using a two-dimensional two-temperature model with different detailed chemical reaction mechanisms that are DRM 19, GRI-Mech 1. 2, and GRI-Mech 3.0. Attention was focused on the influence of these mechanisms on predictions of the temperature distributions in the burner, chemical structure as well as syngas production. The equivalence ratio was a fixed value of 1.5, while the volumetric ratio of CO_2_ to CH_4_ was changed from 0 to 1. The predicted results were compared with the available experimental data. It was revealed that the chemical reaction mechanisms have little effect on the temperature distribution in the burner except for the exothermic zone. It indicted that the smaller kinetic DRM 19 can precisely predict the temperature distributions in the burner, using DRM 19 was recommended to save computational time when the detailed components of the syngas was not taken into consideration. In addition, all the three mechanisms predicted the same trend of molar fraction of CO, H_2_, and CO_2_ with experimental results. Good agreement between the experiment and predictions of major species was obtained by GRI-Mech 1.2 and GRI-Mech 3.0, the two mechanisms had the same accuracy in predicting CO, H_2_, and CO_2_ production. However, computations with DRM 19 under-predicted the molar fraction of CO and H_2_. Furthermore, it was shown that the thermal conductivity of porous media has significant effect on the syngas production. In general, the temperature was increased as the thermal conductivity of the porous media was reduced and the H_2_ production was increased.

## Highlights

- The highest combustion temperature in the reaction zone is predicted by DRM 19
- Computations by DRM 19 under-predict the molar fraction of CO and H_2_
- GRI-Mech 3.0 and 1.2 have the same accuracy in predicting the syngas component;
- Increase in thermal conductivity of solid leads to increase in temperature in the two-layer burner;
- Increase in thermal conductivity of solid leads to increase in H_2_ production.

## Introduction

The interest in syngas production from fuel-rich partial oxidation within inert porous media has increased significantly during the last two decades. This is because this technology combines several positive features, such as quick start-up, fast dynamic response, no need for external heat, steam, and catalyst. Kennedy et al. (1999) and Muhammad (2016) presented detailed reviews of this subject.

In order to improve combustion process and increase syngas production, a large amount of researches have been conducted in recent years and some significant developments have been made (Mujeebu, 2016).

There are many experimental and numerical studies on fuel-rich partial oxidation in porous media burner. In general, there are two design approaches commonly employed for syngas production in porous burner: the transient combustions systems and stationary systems.

For the transient combustion system, the porous media burner was filled with homogeneous porous media and flame propagations were observed either in downstream or upstream direction in most cases. Hydrogen production by transient filtration combustion was extensively studied by Kennedy group (Drayton et al., 1998), they proposed reciprocal flow burner (RFB) for syngas production and the combustion wave was restricted in the burner by periodically changing the direction of the flow. An extremely high flammability limit was extended to an equivalence ratio of eight for CH_4_/Air mixtures. In the experimental and numerical studies of Kennedy et al. (2000), upstream, downstream, or standing wave was observed experimentally, mainly depending on the CH_4_/Air equivalence ratio (φ) for the wide range of 0.2 ≤ φ ≤ 2.5 and the fixed gas velocity of is 0.25 m/s. The experimental results showed that 60% of the methane was converted to CO and H_2_ for φ > 2.

Toledo et al. (2009) studied experimentally the transient combustion system for syngas production in a packed bed filled with 5.6 mm Al_2_O_3_ spheres. It was shown that the maximum hydrogen yield was close to 50% and CO yield was close to 80%. To increase the syngas production, two methods were conducted, namely, adding stream during filtration combustion (Araya et al., 2014), or providing external heat source to the burner system (Zheng et al., 2012a). Experimental and numerical results by Araya et al. (2014) showed that hydrogen yield increases with increasing steam content in methane-air mixture. Several fuels conversion to syngas were experimentally studied by Toledo et al. (2016) and Gonzalez et al. (2018), they (Toledo et al., 2016) studied conversion efficiency of partial oxidation combustion in pellet packed bed for liquefied Petroleum Gas, butane, propane, Diesel fuel, and Heavy Fuel Oil. It was shown that conversion efficiency of Heavy Fuel Oil was highest than other fuels and reached up to 45%.

For the transient combustion system, combustion wave always propagates upstream or downstream and this leads to flame extinction at the end. To stabilize the flame within porous burner, two types of burner, namely, RFB (Drayton et al., 1998) and two-layer burner (Zeng et al., 2017; Wang et al., 2018) filled with different material or structures of the porous media were developed.

For the stationary systems, the burner was filled with different material or structures of porous media, the flame was restricted near two sections of the burners under a certain range of equivalence ratio and gas velocity. Zeng et al. (2017) studied experimentally and numerically the syngas production in a two-layer burner for fuel-rich combustion with a range of CO_2_ content in the CH_4_ fuel. The experimental results showed that the reforming efficiency increased from 39.1 to 45.3% when the CO_2_ was injected into the system. In their subsequent study (Wang et al., 2018), the performance of methane partial oxidation in a two-layer burner filled with alumina pellets of different diameters in the downstream was conducted. According to the highest reforming efficiency, an optimized burner was determined, which was composed of 7.5 mm pellets in the downstream section and 2–3 mm pellets in the upstream section.

For simulation of syngas production from fuel-rich partial oxidation in porous media, it is essential to use detailed or reduced chemistry for investigation of detailed composition of syngas and intermediary components, GRI-Mech combustion mechanism was widely used, which includes detailed Kinetics GRI-Mech 1.2, GRI-Mech 2.11, and GRI-Mech 3.0 (Bowman et al., 2009). For saving computational cost, smaller chemical kinetics like Peters (Mauss and Peters, 1993) or overall mechanism was used by the researchers. Based on the volume-averaged method, one-dimensional or two-dimensional model with these chemical kinetics has been applied to predict the temperature profiles, syngas production, and conversion efficiency.

Kinetic simulations with GRI-Mech 1.2 were conducted and CHEMKIN software was used to solve the chemical reactions (Drayton et al., 1998). Their results indicated that the partial oxidation of methane in porous media occurs ignition and steam reformation processes. Kennedy et al. (2000) analyzed chemical structures of CH_4_/Air mixture in packed bed using one-dimensional model with GRI-Mech 1.2. Their analysis of the reaction pathway showed significant changes of the combustion mechanism from ultra-lean to ultra-rich conditions.

Dhamrat and Ellzey (2006) modeled transient filtration combustion of fuel-rich combustion in the range of equivalence ratio from 1.5 to 5 using a two-dimensional two-temperature model with GRI-Mech 3.0. Special attention was focused on the transient behavior of fuel-rich combustion. It was shown that high solid temperature zone enlarged as combustion wave propagated downstream, which was preferred to steam-reforming reaction and methane conversion efficiency was increased. In addition, they presented the effect of solid properties on the syngas production.

Toledo et al. (2009) modeled syngas production for multiple fuels in porous burner using a one-dimensional two-temperature model with GRI-Mech 3.0. Their predictions showed that fuel-rich partial oxidation in porous media could be used to reform C1-C3 gaseous fuels into hydrogen and syngas. Partial oxidation of methane in a porous reactor was investigated numerically by Zheng et al. (2012a) based on a two-dimensional two-temperature transient model with GRI-Mech 1.2. Results showed that both the gas and solid temperatures increased in the first 400 s and then the variation of maximum combustion temperature was negligible. To increase the CO and H_2_ yields, adding external heat energy for the combustion system was proposed by Zheng et al. (2012b), they modeled partial oxidation of methane in a RFB applying a one-dimensional two-temperature model with GRI-Mech 1.2. Results showed that CO and H_2_ yields increased significantly with the external heater power. An industrial reformer was numerically studied using two-dimensional model with GRI-Mech 3.0 and turbulent effect was taken into account (Xu et al., 2014). Their results showed that the methane conversion efficiency increased as the operating pressure was increased and 3.0 MPa was recommended for industrial operation.

Kostenko et al. (2014) modeled methane partial oxidation in a porous reactor using a one-dimensional two-temperature model with detailed kinetic mechanism including soot formation. Their predictions showed that the steam-soot reaction reduces soot formation and increases hydrogen production. An overall three-step six-component chemical kinetic model was developed for methane partial oxidation in porous media (Dobrego et al., 2008). The parameters of kinetic model were derived by adjusting to the experimental conditions and they suggested that the developed model could be combined with detailed chemistry.

To increase conversion efficiency, Dorofeenko and Polianczyk (2016) proposed a new version of RFB for syngas production, in which the methane-steam and air were supplied separately to the RFB. The air was directly feed into the RFB without preheating, whereas the methane-steam was preheated to high temperature prior to entering into the reaction zone by periodically changing the direction of its flow. Zeng et al. (2017) modeled fuel-rich partial oxidation of CH_4_/CO_2_ mixture in a two-layer burner using a two-dimensional two-temperature model with the detailed chemical kinetic (Peters). It was shown that the predictions of temperatures and major species matched well with the experimental results. Fuel-rich combustion in porous media was studied using skeleton diagrams and sensitivity analysis (Futko, 2003). They suggested that the combustion wave could be divided into three regions that are preheating zone, exothermic zone and endothermic zone, depending on the heat release.

To save computation time, for fuel-lean combustion in porous media, one-step kinetic still plays an important role in understanding the combustion and heat transfer processes using volume-averaged method (Liu et al., 2009; Fan et al., 2017, 2019; Wang et al., 2019). The influence of chemical reaction mechanisms on predictions of the filtration combustion has been conducted for fuel-lean condition (Hsu and Matthews, 1993; Mohammadi and Hossainpour, 2013). Hsu and Matthews (1993) numerically investigated the effect of chemical reaction mechanisms on the CH_4_/air premixed combustion characteristics in porous media under the condition of equivalence ratio <1. They concluded that it is essential to use multistep kinetics for accurate predictions of temperature profiles, species distribution, energy release rate and pollutant emissions. Mohammadi and Hossainpour (2013) simulated the experiment of Trimis and Durst (1996) applying a two-dimensional two-phase model with different four multistep kinetics that are GRI-Mech 3.0 mechanism, GRI-Mech 2.11 mechanism, the skeletal and 17 species mechanism (Peters). They studied the effects of these models on temperature, species profiles and pollutant emissions at the fixed equivalence ratio of 0.67 for CH_4_/Air mixture. Results showed that the four models have the same accuracy in predicting temperature distributions and the difference between these profiles was not more than 2%. In addition, the GRI-Mech 3.0 showed the best prediction of NO emission in comparison with the experimental data.

As reviewed above, a lot of numerical studies on fuel-rich combustion for syngas production in porous media burner have been conducted applying different detailed mechanisms, the influence of these kinetics on prediction of premixed combustion for fuel-lean combustion in porous burner has been revealed. However, it is still not certain the influence of the prediction of syngas production for fuel-rich combustion in porous media using different chemical reaction mechanisms. To save computation time, one may prefer to use a smaller detailed kinetic for modeling of syngas production in porous burner, but it was not known if this mechanism have the same accurate in predicting the combustion characteristics as the more detailed mechanisms. At the same time, one-dimensional model was widely used to save computational time and the two-dimensional study is scarce, which is more accurate to compute the heat loss to the surrounding through burner walls.

The aim of the present work is to investigate the influence of chemical reaction mechanisms on predictions of the temperature profiles, chemical structures and therefore the output of the syngas production in the exhaust gas, using a two-temperature two-dimensional model. In contrast to previous studies which emphasized on the effect of chemical kinetics on fuel-lean combustion in porous media, we explore the syngas production for fuel-rich combustion in porous media.

## Physical Model

**Figure 1:**
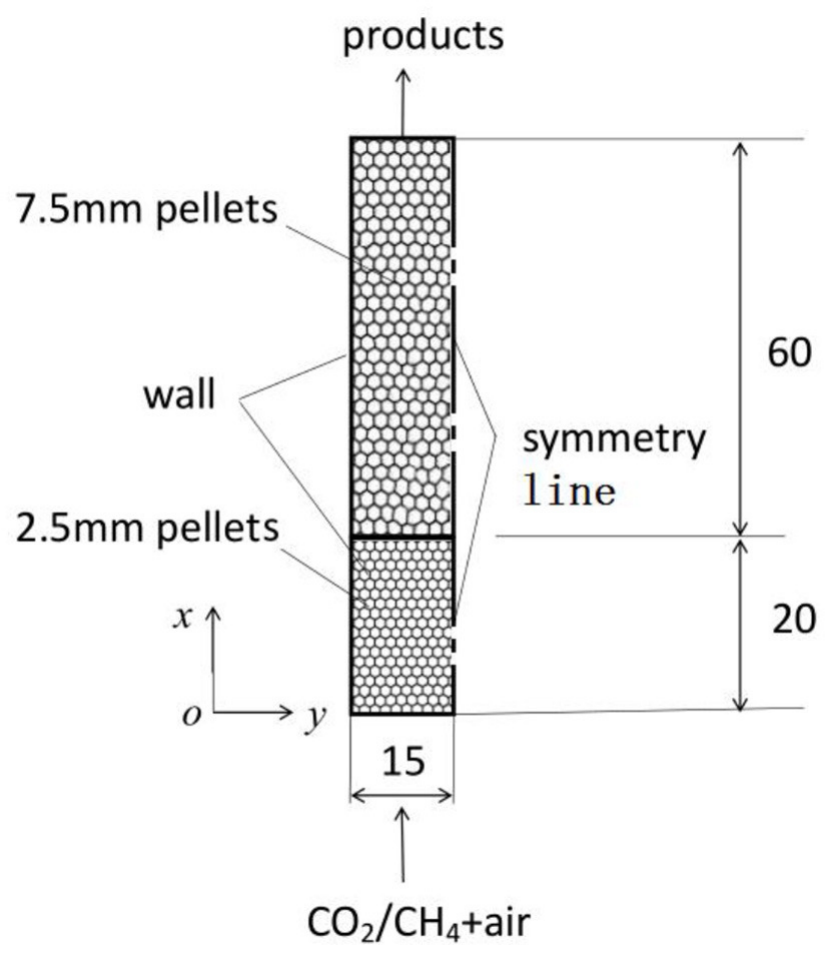
Schematic of the two-layer burner. All dimensions are in mm.

A two-layer porous burner reported by Zeng et al. (2017) is considered in the present work to study the sensitive of different kinetics to the predictions of syngas production in porous media. As shown in Figure 1, the burner was designed to study the effect of CO_2_ addition on the conversion efficiency of CH_4_ partial combustion within packed bed, which consists of two layers of alumina pellets with different diameters. In the upstream section the burner was filled with 2–3 mm alumina pellets that are 20 mm long, while the downstream layer was filled with 7.5 mm alumina pellets with length of 60 mm. The premixed mixture of methane with different amount of CO_2_ and air was feed into the burner and combustion waves were restricted near the interface of the upstream and downstream sections. For simplification, the pellet diameter in the upstream section is assumed to be a constant value of 2.5 mm. 2-D computations are considered in this work to save computational time. To simplify the problem, the following assumptions are made;

1. The alumina pellets are assumed to be inert homogeneous and optically thick media, the solid radiation is taken into account using the effective radiation thermal conductivity model.
2. Gas flow in the packed bed is assumed to be laminar and gas radiation is ignored.
3. The porosity variation near the tube wall is ignored and the thermal conductivity of the packed bed is the same for the two layers.

The chemistry is treated with two detailed kinetics and a reduced chemical mechanism in the computation. These mechanisms include two full mechanisms complied by the Gas Research Institute, namely GRI-Mech 1.2 (32 species, 177 reactions), GRI-Mech 3.0 (53 species, 325 reactions), and DRM 19 (Kazakov and Frenklach, 1994) (20 species, 58 reactions) which is a reduced mechanism based on GRI-Mech 1.2. The gas thermal and transport properties are obtained from the Chemkin and Tranfit packages (Kee et al., 1986).

Under the above assumptions, a set of differential equations can be obtained.

Continuity equation:

(1) $$\begin{array}{l} \nabla \cdot \left(\rho_{g}\text{v}\right)=0 \end{array}$$

where ρ_*g*_ represents the gas density; **v**denotes the velocity vector.

Vertical momentum equation

(2) $$\begin{array}{l} \nabla \left(\rho_{\text{g}}\text{v}u\right)=\nabla \left(\mu \nabla u\right)-\frac{\Delta p}{\Delta x} \end{array}$$

Where *u*represents the vertical velocity; μ is dynamic viscosity, *p* is the pressure.

Horizontal momentum equation:

(3) $$\begin{array}{l} \nabla \left(\rho_{\text{g}}\text{v}\nu \right)=\nabla \left(\mu \nabla \nu \right) \end{array}$$

where *v* denotes the horizontal velocity. The pressure loss in the vertical direction is computed as (Ergun, 1952),

(4) $$\begin{array}{l} \frac{\Delta p}{\Delta x}=150\frac{{\left(1-\varepsilon \right)}^{2}}{\varepsilon^{3}}\frac{\mu u^{\prime }}{d^{2}}+1.75\frac{1-\varepsilon }{\varepsilon^{3}}\frac{\rho_{\text{g}}{u^{\prime }}^{\text{2}}}{d} \end{array}$$

where ε is the porosity of the porous media, *d* is the pellet diameter, *u*′ is superficial velocity and *u*′ = ε*u*.

Gas phase energy equation:

(5) $$\nabla .\left(\rho_{\text{g}}c_{\text{g}}\text{v}T_{\text{g}}\right)=\nabla .\left(\lambda_{\text{g}}\nabla {\text{T}}_{\text{g}}\right)+{\text{h}}_{\text{v}}\left(T_{\text{s}}-T_{\text{g}}\right)+\sum_{\text{i}}\omega_{\text{i}}h_{\text{i}}W_{\text{i}}$$

where *T*_g_, λ_g_, *c*_*g*_ are the gas temperature, thermal conductivity and specific heat, respectively. *T*_s_ denotes the solid temperature; ω_i_, *W*_*i*_ are the chemical reaction rate and molecular weight of species i. *h*_*v*_ is the volumetric convective heat-transfer coefficient between the gas and solid phases (Kaviany, 1994),

(6) $$\begin{array}{l} h_{v}\text{=6}\varepsilon N{\text{u}}_{\text{v}}\lambda_{\text{g}}\text{/}{\text{d}}^{\text{2}},Nu_{v}=2+1.1{\text{Pr}}^{1/3}{\text{Re}}^{0.6} \end{array}$$

where Nu_v_, Pr, and Re is the Nusslet number, Prandtl number, and Reynolds number, respectively.

Solid phase energy equation:

(7) $$\begin{array}{l} \nabla \cdot \left(\lambda_{\text{eff}}\nabla T_{\text{s}}\right)+h_{\text{v}}\left(T_{\text{g}}-T_{\text{s}}\right)=0 \end{array}$$

Where λ_eff_ is the effective thermal conductivity of the porous media and can be expressed as λ_eff_ = λ_*s*_ + λ_rad_, λ_*s*_, and λ_rad_ are the solid thermal conductivity. We assume that the thermal conductivities for the two sections are same and the influence needs to be further studied. The radiative heat transfer coefficient of the alumina pellets, respectively. λ_rad_ is expressed as (Serguei et al., 1996).

(8) $$\begin{array}{l} \lambda_{rad}=\left(32\varepsilon \sigma d/9\left(1-\varepsilon \right)\right){T_{s}}^{3}. \end{array}$$

Species conservation equation:

(9) $$\begin{array}{l} \nabla \cdot \left(\rho_{g}\text{v}Y_{\text{i}}\right)-\nabla \cdot \left(\rho_{\text{g}}\nabla Y_{\text{i}}\right)-\omega_{\text{i}}W_{\text{i}}=0 \end{array}$$

Where *Y*_*i*_ is mass fraction of species i.

## Boundary Conditions

The following boundary conditions are specified in the model:

1. Inlet
  (10) $$\begin{array}{r} T_{\text{g}}=T_{\text{s}}=300\text{K},u=u_{0},v=0\\ Y_{\text{CH}4}={Y_{\text{CH}4}}_{,\text{in}},Y_{O2}={Y_{O2}}_{,\text{in}},Y_{\text{CO}2}={Y_{\text{CO}2}}_{,\text{in}},\\ Y_{\text{N}2}={Y_{\text{N}2}}_{,\text{in}} \end{array}$$
2. Outlet
  (11) $$\begin{array}{l} \frac{\partial T_{\text{g}}}{\partial x}=\frac{\partial T_{\text{s}}}{\partial x}=\frac{\partial \left(Y_{\text{i}}\right)}{\partial x}=0. \end{array}$$
3. Solid temperature at the inlet and outlet;
  (12) $$\begin{array}{l} \lambda_{\text{eff}}\frac{\partial T_{\text{s}}}{\partial x}=-\varepsilon_{\text{r}}\sigma \left({T^{4}}_{s,\text{in}/\text{out}}-{T_{0}}^{4}\right) \end{array}$$
  ε_*r*_ is the solid surface emissivity, σ is the Stefan-Boltzmann constant, *T*_0_ is ambient temperature.
4. At y = 15 mm, symmetry conditions are imposed;
  (13) $$\begin{array}{l} \frac{\partial T_{\text{g}}}{\partial y}=\frac{\partial T_{\text{s}}}{\partial y}=\frac{\partial Y_{\text{i}}}{\partial y}=\frac{\partial u}{\partial y}=v=0 \end{array}$$
5. Wall
  At y = 0 mm, heat loss to the surroundings through the burner walls by convective heat transfer is considered and heat flux $\dot{q}$ is defined as,
  (14) $$\begin{array}{l} \dot{q}=\frac{\lambda }{\delta }\left(T_{\text{wall}}-T_{0}\right) \end{array}$$
  where λ is thermal conductivity of insulation, δ is thickness of insulation.

## Initial Conditions and Solution

The governing equations presented above are numerically solved by a CFD software Fluent 15.0. To allow the gas and solid phases have different temperatures, user defined function and scalars provided by Fluent 15.0 are used to solve a separate energy equation for the solid phase. Radiative and conductive transport through the packed bed, convective heat transfer between the gas and solid phases is taken into account in the model, as shown in the above Equation (7).

The SIMPLE algorithm is used to handle the pressure and velocity coupling. At the downstream, the solid temperature with a thickness of 4 mm is set to be 1,800 K to model the ignition process. Mesh independence of the results are verified. The computational domain is discretized into 600 cells in the upstream section and 3,600 cells in the downstream section. When the solution is converged, the mesh of the reaction zone is densified.

A residual error of 10^−6^ for energy equations and 10^−3^ for all other equations are taken as convergence criteria.

The syngas energy conversion efficiency is defined as:

(15) $$\begin{array}{l} \eta_{e-s}=\frac{Y_{\text{H}2}\times LHV_{\text{H}2}+Y_{CO}\times LHV_{\text{CO}}}{Y_{\text{CH}4,\text{in}}\times LHV_{\text{CH}4}} \end{array}$$

where LHV_H2_, LHV_CO_, LHV_CH4_ are the low heating value of H_2_, CO, and CH_4_, respectively. Table 1 presents the symbol used in this work.

**Table 1:** Symbols used in this work.

**Nomenclature**	
*d* diameter of spheres, m	*h_*i*_* the molar enthalpy of species i, KJ/Kg
*h_*v*_* convective heat transfer between the solid and gas phases, W/m^3^ K	*p* pressure, Pa
*T* temperature, K	*T*_0_ ambient temperature, K
*u* vertical velocity, m/s	*v* horizontal velocity, m/s
*W_*i*_*molecular weight of species i, K_g_/*K*_*mol*_	*x* vertical coordinate, m
*X* molar fraction	*y* horizontal coordinate, m
*Y* mass fraction	
**Greek symbols**	
φ equivalent ratio	λthermal conductivity, W/m K
λ_eff_ effective thermal conductivity, W/m K	λ_rad_radiation conductivity, W/m K
ρ density, Kg/m^3^	ε porosity
ω_i_reaction rate of species i, K_g_/*K*_*mol*_	σ Stephan-Boltzmann constant, W/*m*^2^ K^4^
α volume flow ratio between CO_2_ and CH_4_	δ Thickness of insulation, m
μ dynamic viscosity, Pa_s_	η_e−s_ syngas energy conversion efficiency
ε_r_solid surface emissivity	
subscripts	
g gas	gayu s solid

## Results and Discussion

### Temperature Distributions

In the experiment (Zeng et al., 2017) the equivalence ratio and air flow rate are fixed while the ratio (α) between the CO_2_ and CH_4_ is changed from 0 to 1. The solid thermal conductivity of alumina is 7.22W/*m*▪*K*at *T* = 1,000 K, according to reference by Munro (1997). For volume average method used in this work, the thermal conductivity of packed bed is defined as 0.04 times of the solid thermal conductivity at *T* = 1,000 K as reference, this means that thermal conductivity of the packed bed is 0.2888 W/m·K unless otherwise stated. Table 2 shows the simulation cases carried out in this work. In the computation, for all computed cases the equivalence ratio is set to be a fixed value of 1.5 and the thermal conductivity of packed bed is varied due to uncertainty of its values. In the following, the temperature reported is along the centerline of the burner.

**Table 2:** Simulation cases carried out in this work.

Equivalence ratio	1.5
The ratio of CO_2_/CH_4_ and its corresponding inlet velocity (m/s)	0 (0.1365), 0.25 (0.1412), 0.5 (0.1458), 1 (0.1551)

**Figure 2:**
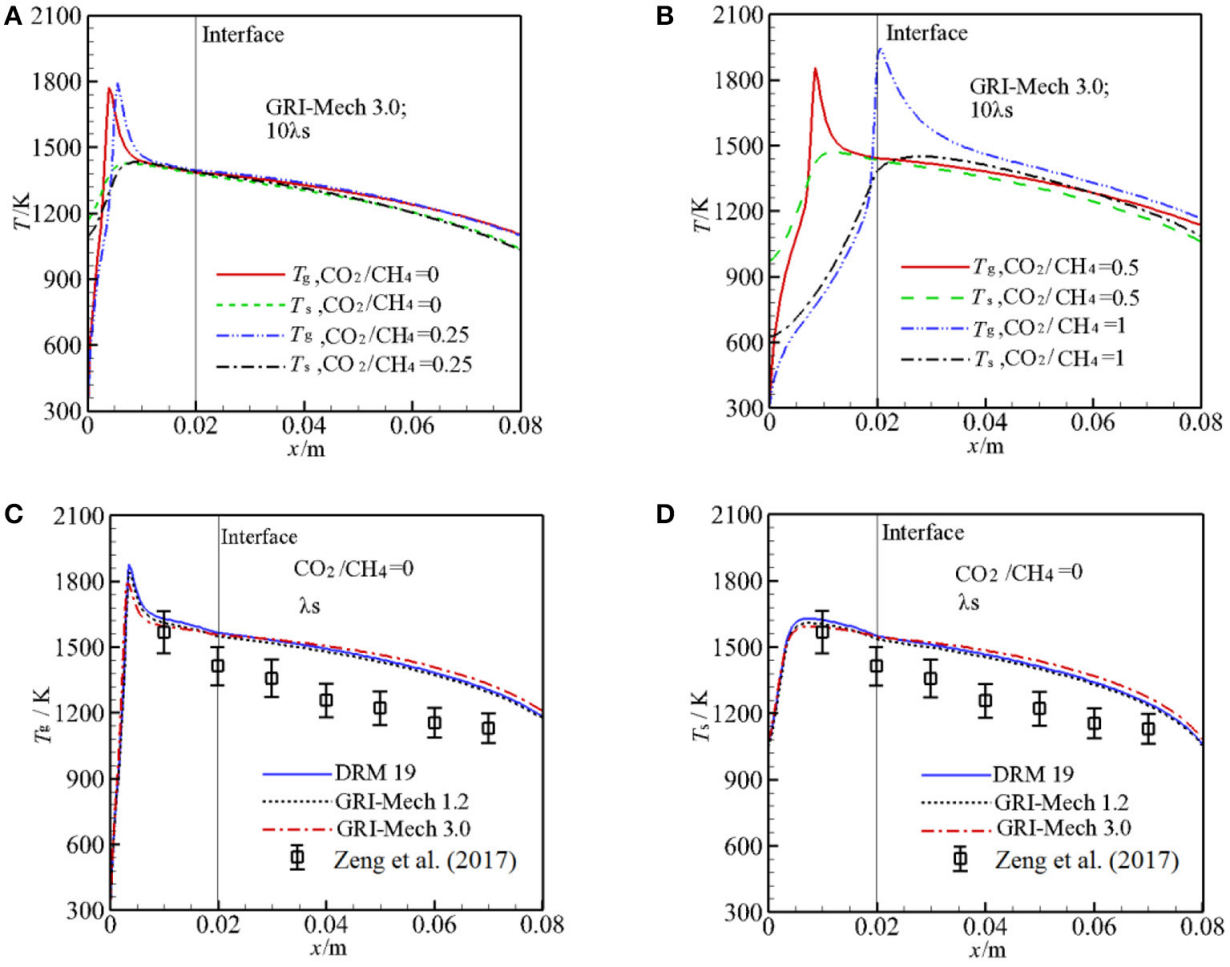
Temperature distributions along the centerline of the burner for different kinetics. **(A)** Gas, solid temperature for molar ratio of CO_2_/CH_4_ = 0, 0.25, and 10 times of thermal conductivity of packed bed with GRI-Mech 3.0; **(B)** Gas, solid temperature for molar ratio of CO_2_/CH_4_ = 0.5, 1, and 10 times of thermal conductivity of packed bed with GRI-Mech 3.0; **(C)** Gas temperature for molar ratio of CO_2_/CH_4_ = 0 with three different mechanisms; **(D)** Solid temperature for molar ratio of CO_2_/CH_4_ = 0 with three different mechanisms.

Figure 2 illustrates the predicted *T*_g_ and *T*_s_ at the centerline (y = 0 mm) for different α as well as experimental values for comparison. Figures 2A,B shows predicted *T*_g_, *T*_s_ with GRI-Mech 3.0 at λ_s_ = 2.888 W/m·K. The calculated results show that the flame shifts from upstream of burner to the interface of two sections as α is increased from 0 to 1. One may expect that the maximum combustion temperature decreases with α due to injection of greater amount of CO_2_ to the burner, but the oppose trend is observed as shown in Figures 2A,B. One of the reasons for this phenomenon is due to the different flame stabilization positions where *h*_v_ are different. *h*_v_ is getting greater as the pellet diameter is decreased, as shown in Equation (6). At the reaction zone, the reaction heat is redistributed through convective heat transfer between the two phases. When the flame is stabilized near the interface, the convective heat transfer weakens and thus the temperature difference between the two phases is enlarged, which results to higher gas combustion temperature in the reaction zone for the smaller pellet diameter.

Figures 2C,D show the predicted *T*_g_, *T*_s_ by three different kinetics for α = 0 at λ_s_ = 0.2888 W/m·K as well as experiment results (Zeng et al., 2017) for comparison. For α = 0, it can be seen that the predicted *T*_g_, *T*_s_ are similar through the burner for the different three kinetics and the temperature differences predicted by the three kinetics are quiet small except for the reaction zone. In the pre-heat zone, the predicted *T*_g_ is almost independent of the mechanisms used. Small difference is observed downstream the reaction zone. The more detailed mechanism is used, the greater peak temperature is obtained in the computation as shown in Figure 2C. In the reaction zone the combustion temperature by the DRM 19 is highest and reaches up to 1,879 K, *T*_g_ by GRI-Mech 3.0 is lowest and the value is 1,786 K. In a word, the temperature distributions in the pre-heat and post reaction zone are almost independent of the mechanisms used, in the reaction zone the peak temperature predicted by different kinetics is rather small. Considering the computation cost and time, choosing a smaller mechanism for predicting temperature distribution in the burner may be a good choice and rather accuracy can be obtained, when the detailed components of the syngas is not taken into consideration. It is noted that the predicted *T*_g_ and *T*_s_ by the three kinetics are always greater than the corresponding experiment values, in the following this deviation will be discussed.

**Figure 3:**
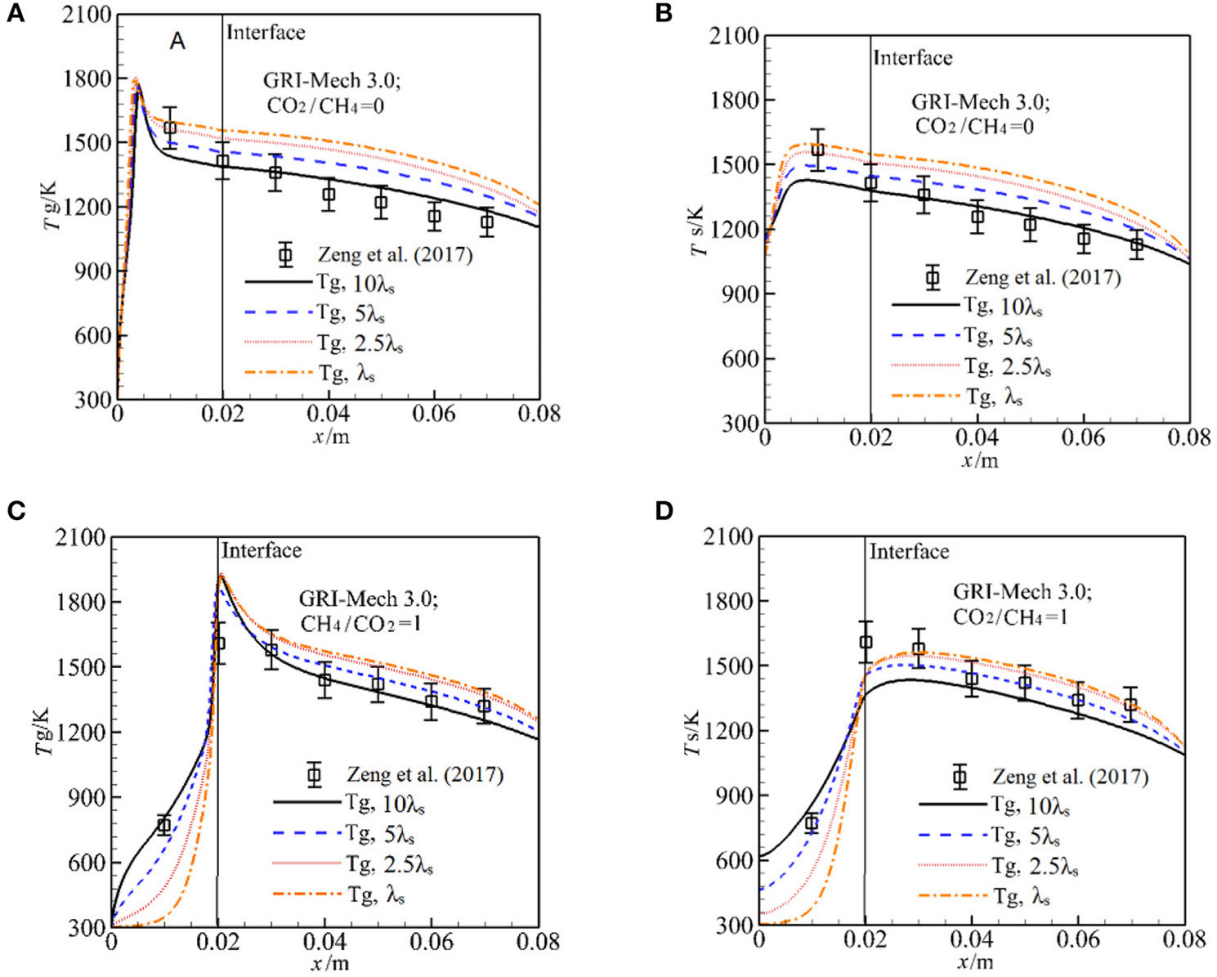
Effect of the packing bed thermal conductivity on gas and solid temperature with GRI-Mech 3.0 for molar ratio of CO_2_/CH_4_ = 0, 1. **(A)** Gas temperature for different solid thermal conductivities at CO_2_/CH_4_ = 0; **(B)** Solid temperature for different solid thermal conductivities at CO_2_/CH_4_ = 0; **(C)** Gas temperature for different solid thermal conductivities at CO_2_/CH_4_ = 1; **(D)** Solid temperature for different solid thermal conductivities at CO_2_/CH_4_ = 1.

In the experimental study of Zeng et al. (2017), λ_s_ was not presented and the effect of λ_s_ on the syngas production was not clear. We test the sensitive of λ_s_ to *T*_g_ and *T*_s_ by increasing λ_s_ by 10 times with other parameters are fixed. The results with GRI-Mech 3.0 for α = 0 are shown in Figures 3A,B. One can see that λ_s_ has significant influence on the temperature distribution in the burner. For α = 0, the flame is stabilized near the burner inlet. The temperatures both for gas and solid phases in the entire burner decrease with λ_s_. This is because an increase in λ_s_ leads to enhance heat condition in the solid phase, more heat is conducted through the solid phase, thus the temperature gradient is getting lower with λ_s_. According to the Equation (12), the heat loss to the surrounding through burner inlet and outlet is increased as λ_s_ is increased, which leads to decrease in temperature in the burner. The predictions with 10 λ_s_ match well with experimental results and the predictions are greater than the experimental value as λ_s_ is decreased.

For α = 1, as shown in Figures 3C,D, the flame stabilizes just downstream the interface, *T*_s_ distribution in the pre-heat zone is oppose to that for α = 0, *T*_s_ is increased with λ_s_. This is attributed to the fact that the heat recirculation from the high solid temperature zone to the downstream direction is enlarged when λ_s_ is increased, which leads to increase in *T*_s_ in the first section of the burner, thus the gas mixture is effectively preheated and *T*_g_ is increased.

### Chemical Structure

**Figure 4:**
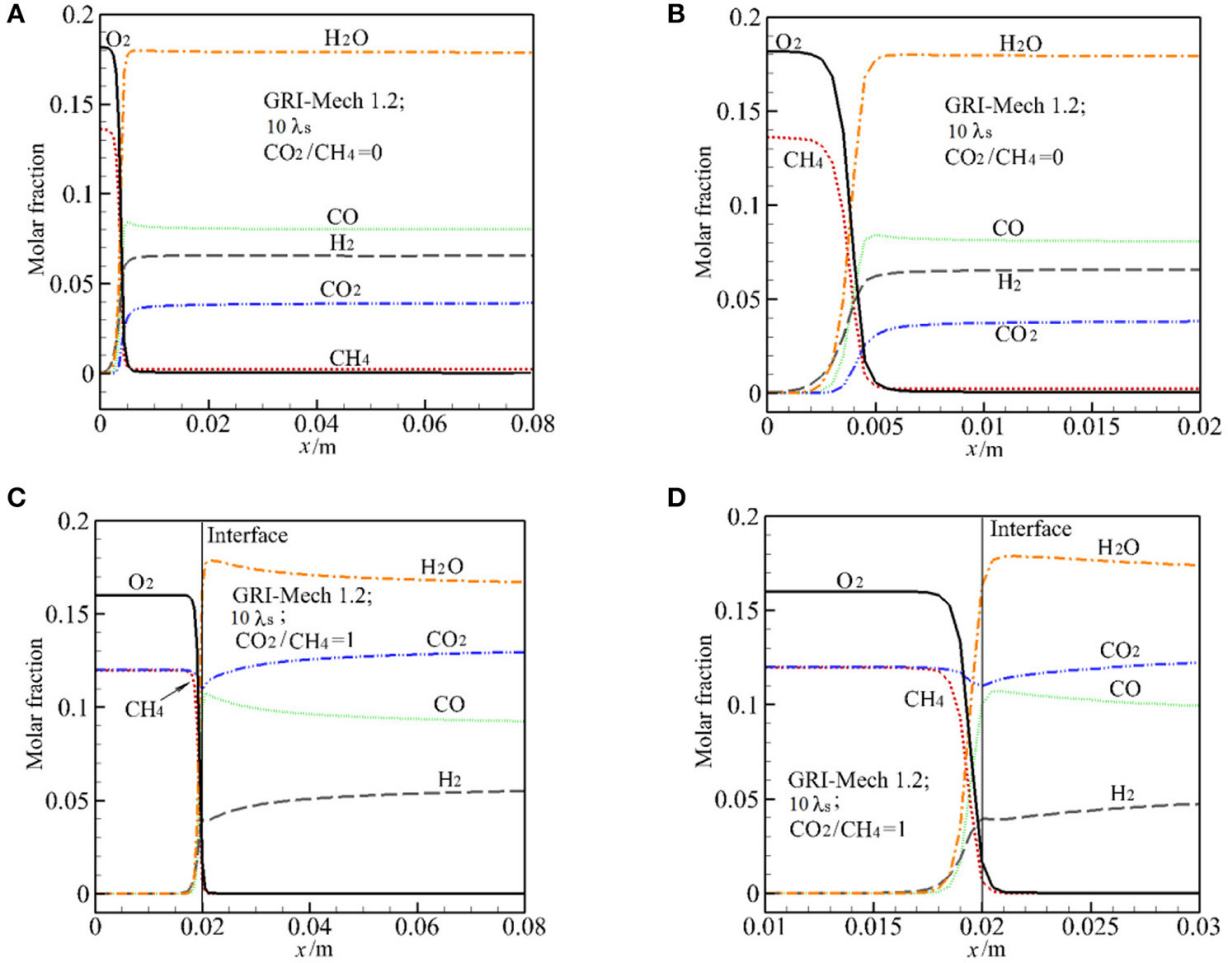
Distribution of major species (molar fraction) by GRI-Mech 1.2 along the centerline and near the reaction zone for molar ratio of CO_2_/CH_4_ = 0 and 1 at 10 times of thermal conductivity of packed bed. **(A)** Molar fraction of major species for CO_2_/CH_4_ = 0; **(B)** Molar fraction of major species at the reaction zone for CO_2_/CH_4_ = 0; **(C)** Molar fraction of major species for CO_2_/CH_4_ = 1; **(D)** Molar fraction of major species at the reaction zone for CO_2_/CH_4_ = 1.

The major species in the exhaust gases includes H_2_, H_2_O, CO, CO_2_, and CH_4_. Consistent with the experiment (Zeng et al., 2017), the Figure 4 show the predicted species based on the wet base. Figure 4 presents the predictions of molar fraction of H_2_O, O_2_, H_2_, CO_2_, CO, CH_4_ by GRI-Mech 1.2 along the vertical direction for α = 0, 1 at 10 λ_s_. For visible, the major species near the reaction zone is also presented in the Figure 4. According to Futko (2003), the combustion wave is composed of the preheating zone, an exothermic zone with partial combustion of methane in the reaction *CH*_4_+0.5O_2_ = CO+2H_2_(16), and an endothermic zone characterized by the reforming processes *C*O+H_2_O = CO_2_+H_2_(17),*C*H_4_+H_2_O = CO+3H_2_(18). For α = 0, it can be seen that extensive reaction occurs in a small region with length about 3 mm near the burner inlet. Methane begins to break down near the burner inlet, CH_4_ and O_2_ are quickly consumed in the exothermic zone. As shown in Figures 4A,B, all the syngas components almost peak after the exothermic zone in this case, only small change in major species is observed due to the reforming reaction. This indicates that the reaction (18) and (19) contribute little to the CO and H_2_ production in the endothermic zone in this case.

For α = 1, the predicted distribution of major species is similar to that of α = 0, except for the distribution of CO_2_ in the exothermic zone. In this zone *X*_H2_ and *X*_CO_ increase sharply and then *X*_H2_ increases slowly while *X*_CO_ reduces slightly in the endothermic zone. As shown in Figures 4C,D, *X*_CO2_ decreases first in the reaction zone, this means that CO_2_ reacts and is consumed. Then *X*_CO2_ continually increases along the vertical direction. It can be seen from Figures 4A,B that, after the exothermic zone the variation rate of the major species for α = 1 is greater than that for α = 0. This is the effect of CO_2_ injection in the burner and also the effect of reforming reaction (17) and (18).

### Major Species in the Exhaust Gases and Conversion Efficiency

**Figure 5:**
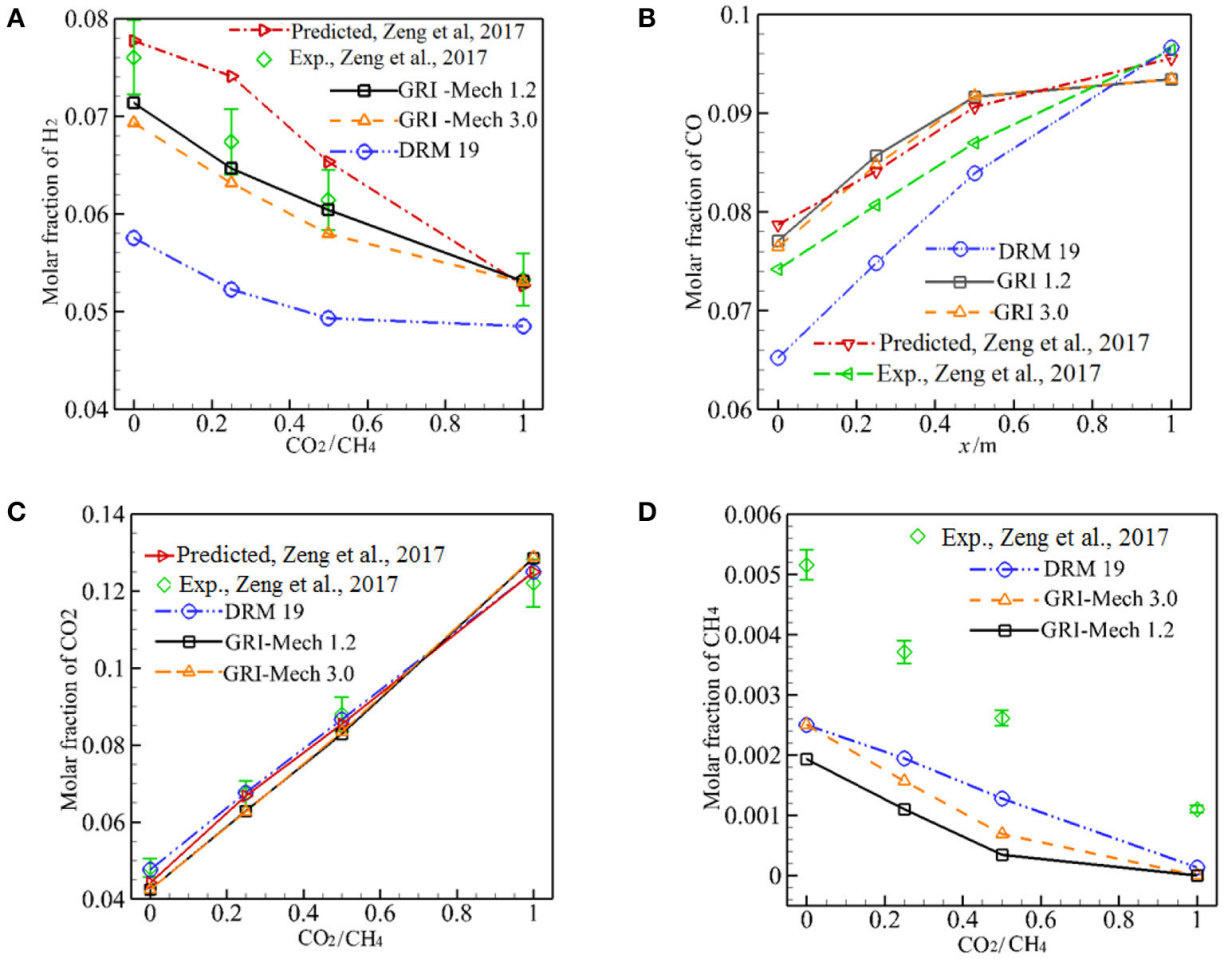
Predicted molar fraction of H_2_, CO, CO_2_, and CH_4_ by three different kinetics. **(A)** Molar fraction of H_2_; **(B)** Molar fraction of CO; **(C)** Molar fraction of CO_2_; **(D)** Molar fraction of CH_4_.

Figure 5 depicts the predicted major species of H_2_, CO, CO_2_, CH_4_ in the exhaust gases as a function of α for three different kinetics at λ_s_ = 0.2888 W/m·K. In Figure 5 the experimental and computational results with Peters by Zeng et al. (2017) are also shown for comparison. As shown in Figure 5A, both the experimental results and predictions show that *X*_H2_ decreases with increasing α from 0 to 1 and the predicted *X*_H2_ by three different kinetics are lower than the experimental values, whereas the computational results by Zeng et al. (2017) are greater than the experimental values. The predicted values by GRI-Mech 1.2 and GRI-Mech 3.0 match well with the experiment when the measurement error is taken into account. The prediction by DRM 19 also shows the same trend with experiment, but its values are significantly lower than the experimental values. For α = 1, the calculated results by GRI-Mech and Peters precisely predict the experimental results, this may be due to the dilution effect of the CO_2_ in the fuel.

Figure 5B shows the comparison of predicted *Y*_CO_ with experimental results and Zeng et al. (2017) for three different kinetics. The predictions by GRI-Mech 1.2 and GRI-Mech 3.0 are greater than the corresponding experimental results, as shown in Figure 5B, Qualitative agreement between the numerical and experimental results can be noted when the measurement error is taken into consideration. It is noted that the predictions by DRM19 deviates significantly from experimental results.

As demonstrated in Figures 5A–C, *X*_CO_ increases while *X*_H2_ decreases with α. Meantime, *X*_CO2_ increases with α, although part of the CO_2_ reacts and is consumed in the exothermic zone as reactant when CO_2_ was injected into the burner. The reaction (17) is an important reformation reaction in the endothermic zone and the injection of CO_2_ into the burner may promote the inverse reaction (17), this may be responsible for the continue increase in *X*_CO_ and decrease in *X*_H2_ when α is increased from 0 to 1.

Figures 5C,D presents predictions of *X*_CO2_, *X*_CH4_ by GRI-Mech, DRM19, and Peters (Zeng et al., 2017). All the combustion models precisely predict *X*_CO2_ for 0 ≤ α ≤ 1. Although all combustion models predict the trend of *X*_CH4_ with α, but the predictions are about two times of the corresponding experimental values.

**Figure 6:**
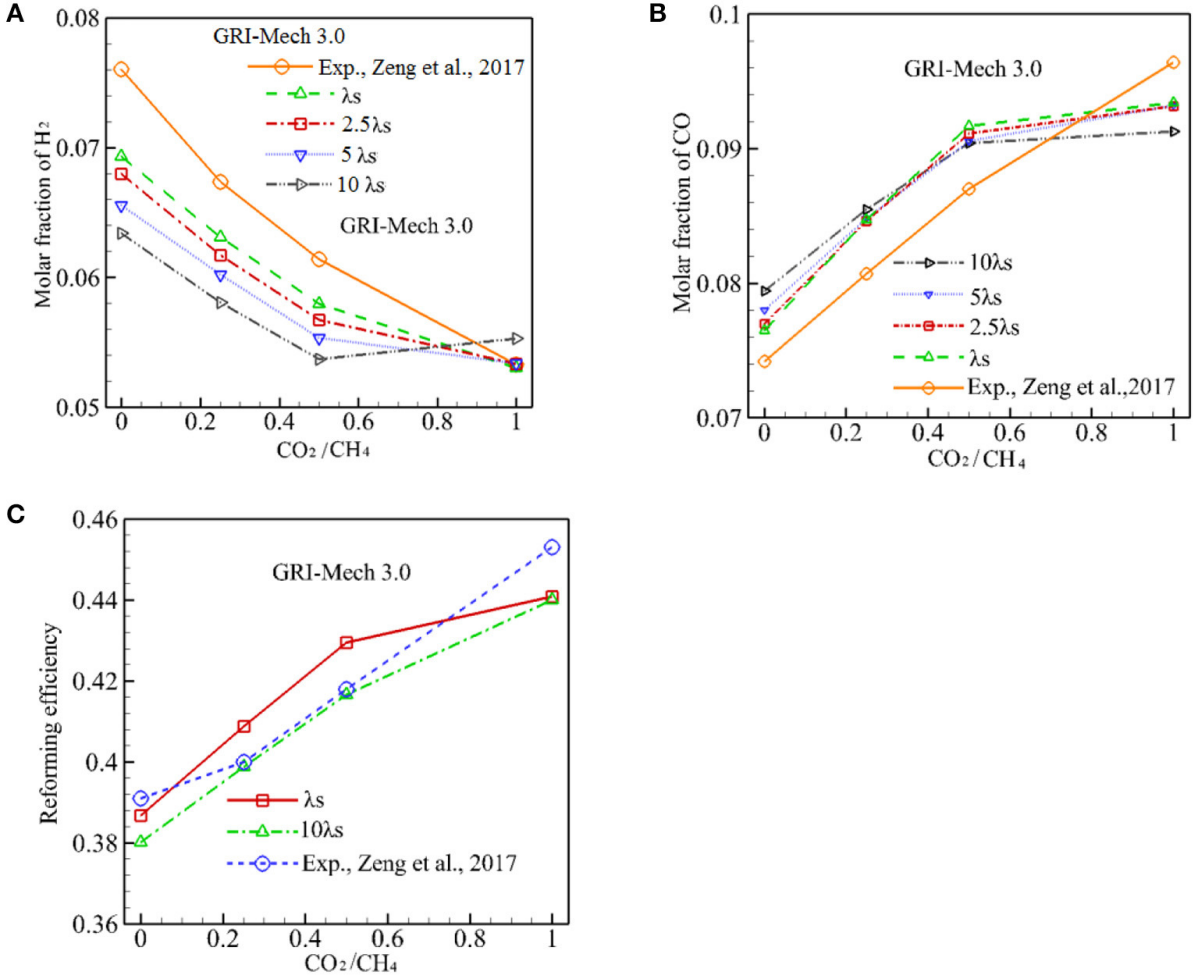
Effect of packing bed thermal conductivity on molar fraction of CO, H_2_, and conversion efficiency with GRI-Mech 3.0. **(A)** Molar fraction of H_2_; **(B)** Molar fraction of CO; **(C)** Reforming efficiency.

To test the effect of λ_s_ on conversion efficiency, λ_s_ is varied from 0.2888 W/m·K to 2.888 W/m·K and the computation is conducted with GRI-Mech 3.0. These results are presented in Figure 6. As discussed above, a lower λ_s_ serves to reduce heat conduction through the solid phase and it is advantageous to form a high solid temperature zone around the reaction zone, thus results in a higher combustion temperature. The increased temperature drives the chemical kinetic to yield a higher percentage conversion. As shown in Figure 6A, *X*_H2_ always increases when λ_s_ is reduced for α ≤ 0.5. However, the effect of λ_s_ on the production of H_2_ diminishes for α = 1. The effect of λ_s_ on the production of CO is shown in Figure 6B, in which it can be seen that increasing in λ_s_ results in a small increase in *X*_CO_ for α ≤ 0.25. Then the oppose trend is observed for α > 0.25, *X*_CO_ decreases with α. A combined effect of on the syngas production is shown in Figure 6C, one can see that a decrease in λ_s_ results in a very small increase in the percentage conversion. For example, the conversion efficiency increases from 41.66 to 42.95% as λ_s_ is decreased from 2.888 to 0.2888 W/m·K for α = 0.5, thus the increase in conversion efficiency can be ignored.

## Conclusions

The influence of chemical mechanisms on the syngas production for rich CO_2_/CH_4_ and air mixture combustion in a two-layer porous burner is investigated using a two-dimensional two-temperature model with GRI-Mech 1.2, GRI-Mech 3.0 and a reduced mechanism (DRM19) based on GRI 1.2. In the computation, the equivalence ratio is a fixed value of 1.5 while the ratio of CO_2_ to CH_4_ is changed from 0 to 1. The sensitive of predicted temperature distributions in the burner and major species in the exhaust gases to the mechanisms used in the model is conducted. The major conclusions from the present study are as follows;

1. Kinetic has no obvious influence on the temperature profiles in the burner expect for the narrow exothermic zone. The predicted temperature distributions by the three kinetics match well with experimental results. For predictions of temperature profiles without consideration of major species, using DRM 19 is recommended to save computational time.
2. The predicted major species (H_2_, CO, CO_2_) by GRI-Mech 1.2 and GRI-Mech 3.0 indicates that the two mechanisms have almost the same accuracy in predicting detailed components, little difference is observed for the whole investigated range. Thus, when the NOx emission is not focus of the study, GRI-Mech 1.2 is recommended to save computational time. However, the predicted molar fraction of H_2_ and CO by DRM 19 is under-predicted compared to experimental values. The three kinetics over-predicted the molar fraction of CH_4_ by a factor about two times of the experimental values.
3. Thermal conductivity of the porous media used in the burner has significant effect on predicting the syngas productions. Increase in the thermal conductivity leads to a decrease in the combustion temperature, and thus increases in H_2_ and decreases in CO, a very small increase in conversion efficiency is observed when the thermal conductivity is decreased by a factor of 10 times and this effect can be ignored.

## Data Availability Statement

All datasets generated for this study are included in the article/supplementary material.

## Author Contributions

JS, MM, and HL conceived and designed the study. YoL analyzed the data. YaL and YD wrote the manuscript.

### Conflict of Interest

The authors declare that the research was conducted in the absence of any commercial or financial relationships that could be construed as a potential conflict of interest.
